# Candidate prognostic factors of presenteeism among French workers: an exploratory longitudinal study

**DOI:** 10.1186/s12889-025-26020-w

**Published:** 2026-01-07

**Authors:** Florian Barbier-Cazorla, Arnaud Lardon, Yolande Esquirol

**Affiliations:** 1 UMR-1295 CERPOP, Université Toulouse-INSERM, Toulouse, France; 2 Occupational and Preventive Medicine, CHU Toulouse, Toulouse, France; 3 Institut Franco-Européen de Chiropraxie, Ivry-sur-Seine, 94200 France

**Keywords:** Presenteeism, Prognostic factors, Occupational health, Psychosocial work conditions, Musculoskeletal disorders, PROGRESS framework

## Abstract

**Background:**

Presenteeism, defined as working despite health problems, represents a major economic and public health burden. While many determinants have been proposed, robust prognostic evidence remains limited. According to the PROGRESS framework, exploratory studies are essential to identify candidate factors before confirmation. This study aimed to explore potential prognostic factors of presenteeism among a sample of French workers.

**Methods:**

A 12-month prospective longitudinal study was conducted in three companies. Adult employees completed baseline sociodemographic characteristics, physical activity, sedentary time, psychosocial work factors, mental health, and musculoskeletal symptom questionnaires. Presenteeism was measured at a follow-up (FU) using the Stanford Presenteeism Scale-6 (SPS-6). High presenteeism was defined as a score of 16 or higher. Logistic regression analyses, adjusted for age, sex, and compliance with physical activity guidelines, were used to identify possible associated factors. Restricted cubic spline analyses were tested for potential nonlinear relationships.

**Results:**

Among the 224 included participants at the baseline, 139 (62.1%) completed the follow-up. The mean age was 42.9 years (± 10.8) and 60.7% were women. Twenty-nine participants (20.9%) presented high presenteeism at a follow-up. Possible associated factors included health care work sector (OR = 13.96, CI 95% 1.73–112.99), blue-collar occupation (OR = 3.91, CI 95% 1.39–11.01), extended workday (10 h or more) (OR = 0.17, CI 95% 0.05–0.60), low sedentary time (OR = 3.93, CI 95% (1.41–10.96), low decision latitude (OR = 5.71, CI 95% 2.23–14.58), job strain (OR = 5.00, CI 95% 1.25–20.08), depression (OR = 3.56, CI 95% 1.30–9.60), poor sleep quality (OR = 1.14, CI 95% 1.01–1.27), and a greater number of painful sites (OR = 1.34, CI 95% 1.05–1.70). Nonlinear effects were observed for sleep disturbances and multisite musculoskeletal pain.

**Conclusion:**

This exploratory study highlights a clustered pattern of psychosocial work, working, and clinical factors associated with presenteeism. In line with PROGRESS recommendations, these findings should be interpreted as hypothesis-generating and require replication/confirmation in larger, independent cohorts. Such work may inform future prognostic models and guide occupational health interventions.

**Supplementary Information:**

The online version contains supplementary material available at 10.1186/s12889-025-26020-w.

## Background

Workplace health promotion has grown significantly over the past decades, evolving from simple ergonomic adjustments to a comprehensive, person-centred and integrative occupational health model that embeds risk evaluation, proactive support measures, and systematic strategies to support both return-to-work and work retention within regular organisational practice [1]. Within this broader context, presenteeism, employees attending work despite health impairments, has emerged as a crucial yet insufficiently addressed issue. Estimates of employer and population datasets indicate that productivity losses attributed to on-the-job impairment can meet or exceed those linked to absenteeism, escalating financial burdens for companies and social welfare systems [2]. Moreover, beyond economic impacts, presenteeism can compromise work quality, increase workplace injury risk, and contribute to downstream disability [1, 2].

Current evidence highlights the complexity of presenteeism, identifying multiple contributing factors. Within the work-related domain, García-Iglesias et al. (2023) and Miraglia & Johns (2016), in systematic reviews, found consistent associations between high presenteeism and psychosocial stressors and managerial pressure in pandemic-era work settings [3, 4]. These findings are echoed in a scoping review by Nowrouzi-Kia et al. (2024), which shows that telework environments, particularly those with low social support or high stress, are conducive to increased presenteeism [5]. Previous evidence by Böckerman & Laukkanen (2010) demonstrated that working-time arrangements, extended weekly hours, mismatches between desired and actual schedules, and worker replaceability were significantly associated with sickness presenteeism among Finnish employees [6].

From a biomedical perspective, various health conditions, particularly those involving chronic pain, sleep disturbances or mood disorders, may also contribute to reduced work performance and presenteeism. For example, musculoskeletal disorders (MSDs) remain a common occupational concern, and recent studies have shown that pain severity, sleep disturbances and functional limitations are correlates of productivity loss and delayed return-to-work [7]. However, recent contributions, including longitudinal studies of insomnia and work performance [8], prospective analyses of sleep disorders in young workers [9], and large cross-sectional assessments of the workday versus non-workday sleep duration [10] still rely on heterogeneous presenteeism measures and do not provide consistent prognostic inference.

To move beyond simply observing which factors are linked to presenteeism and instead build reliable knowledge that helps predict who is most at risk, prognostic factor research offers a valuable methodological framework. Specifically, the PROGnosis RESearch Strategy (PROGRESS, Part 2) sets out standards for recognising and evaluating factors that forecast future outcomes, facilitating targeted interventions [11]. Applying this structured prognostic approach to presenteeism could be particularly beneficial, as accurate prognostic indicators would allow timely interventions, tailored prevention strategies, and optimised allocation of workplace resources.

Current research into presenteeism remains predominantly exploratory. Recent reviews highlight major methodological heterogeneity, including varying definitions, and the use of multiple measurement tools (e.g., SPS-6 vs. HPQ/WLQ). In addition, most available studies rely on cross-sectional designs and context-specific samples [1, 3, 5]. Such designs limit the ability to identify prognostic factors. For this reason, evidence remains insufficient to support a formal replication or confirmation phase within the PROGRESS framework. Early-stage longitudinal exploratory prognostic studies are therefore required to establish which factors warrant future confirmation. Given this current state of evidence, the field aligns with the exploratory phase described in PROGRESS-2, which emphasises the hypothesis generation rather than confirmatory prognostic inference.

Identifying a broad yet plausible set of work factor and biomedical predictors creates the foundation for future longitudinal modelling and causal investigations. Ultimately, determining whether these factors are modifiable, and how they interact, will be essential for designing effective, evidence-informed workplace interventions.

Thus, this 12-month exploratory prospective study among workers aims to identify candidate prognostic factors (related to working conditions, and pathology such as musculoskeletal disorders, mental health or sleep quality) associated with presenteeism in the workplace, evaluated with a referenced questionnaire (Koopman et al., 2002; Lauzier et al., 2019).

## Methods

### Study design

This longitudinal exploratory study followed French workers and is reported according to the STROBE recommendations for observational studies [12]. The STROBE checklist is available in Additional File 1.

### Ethics approval and consent to participate

The French longitudinal exploratory study was approved on October 5, 2021, by the Ethics Committee for the Protection of Persons of Ile de France V in France [000011–06102021185740. Cat 3 HPS]. Formal electronic informed consent was obtained from all participants before any data collection through a two-step electronic and oral consent procedure. Specifically, all potential participants received comprehensive written information about the study, including: (1) study objectives and procedures; (2) expected duration of participation (baseline and 12-month follow-up); (3) potential risks and benefits of participation; (4) data confidentiality and security measures; (5) voluntary nature of participation; and (6) the right to withdraw from the study at any time without penalty or loss of benefits. Participants were given adequate time to review this information and ask questions before providing their consent. Informed consent was documented electronically via the Castor EDC platform, where participants actively confirmed their understanding and agreement to participate in checking consent boxes. Oral consent procedures were conducted during on-site recruitment sessions where study staff verbally explained the study and answered participant questions before directing them to the electronic consent form. All procedures in this study adhered to the ethical standards of the institutional and national research committees and to the principles of the Declaration of Helsinki (World Medical Association, 2013).

### Study participants and setting

The sample was assembled in collaboration with company managers and occupational health teams that partnered voluntarily with the research team.

A total of three private-sector companies (manufacturing, health care and higher education) participated, with more than one hundred workers for each one and located in three different French regions.

Recruitment was therefore voluntary and multicentric, allowing inclusion of heterogeneous work environments while remaining logistically feasible for the 12-month longitudinal follow-up.

Baseline and follow-up results on workers were collected between June 2022 and December 2024.

### Eligibility criteria

To be eligible, participants had to be adults capable of providing independent informed consent (i.e., not under guardianship, legal protection, or minor status), employed on a permanent contract over the course of the study and affiliated with a social security system or a beneficiary of such a system. The participants had to have a written comprehension of French.

The exclusion criteria were adults under legal protection or those who were unable to provide informed consent.

### Procedures

All companies expressing interest and having eligible workers were included. The information notices detailed objectives, risks, benefits, and participant tasks. All eligible workers were invited to complete a baseline questionnaire bank and a short 12-month follow-up. Recruitment ran from June 2022 (first company) to December 2023 (last company). Follow-up data were collected between June 2023 and December 2024.

During on-site recruitment sessions, trained research staff provided additional oral explanations and answered individual questions. Participants were informed that their decision to participate or not would have any impact on their employment status or relationship with their employer. Only after receiving all necessary information and having their questions answered were participants directed to the detailed information notices. These notices provided comprehensive information about the study purpose, methodology, time commitment, voluntary nature of participation, confidentiality protections, and contact information for the research team for any questions or concerns. The electronic consent form required participants to actively confirm their understanding of key study elements before proceeding to the baseline questionnaires. Participants were reminded of their right to withdraw at the beginning of the follow-up phase.

### Data collection

At inclusion, participants completed a baseline assessment via the Castor Electronic Data Capture (Castor EDC) platform consisting of: (1) a brief sociodemographic questionnaire developed specifically for this study, and (2) six previously validated and published questionnaires (The Stanford Presenteeism Scale-6, the Global Physical Activity Questionnaire, the Job Content Questionnaire, the Hospital Anxiety and Depression Scale, the Pittsburgh Sleep Quality Index and the Nordic Musculoskeletal Questionnaire).

Follow-up questionnaires were distributed via Castor EDC 12 months later and consisting of: (1) a brief sociodemographic questionnaire developed specifically for this study, and (2) two previously validated and published questionnaires (The Stanford Presenteeism Scale-6, and the Nordic Musculoskeletal Questionnaire).

A short sociodemographic questionnaire was developed for this study to collect basic participant characteristics including age, gender, weight, height, job title (for socioprofessional category classification according to INSEE nomenclature), and years of professional experience. These items used standard demographic questions commonly employed in occupational health research. Complete baseline and follow-up questionnaires were provided as Additional File 2 and Additional File 3.

All health-related and work-related variables were assessed using previously validated and published questionnaires. No new health or work assessment tools were developed for this study. All validated questionnaires were administered in their complete, unmodified forms using the published scoring algorithms. Detailed information about each instrument, including specific scoring procedures and cut-off values, is provided in the respective subsections below and summarised in Table 1.

**Table 1:** Description and role of study variables

Variable name	Variable role	Type of variable	Definition	Question (measure)
*Individual’s factors*				
Age	Adjustment	Continuous	Age of participants	Age (years)
Gender	Adjustment	Categorical	Gender of participants	Gender (women/men/other) (Ref: [women])
Leisure Physical activity	Adjustment	Categorical	This variable indicates whether the participant meets the World Health Organization (WHO) recommendations for leisure physical activity, defined as engaging in at least 150 min of moderate-intensity activity per week, spread over at least 5 days or 75 min of vigorous-intensity activity per week, spread over at least 3 days.	Section 3 Leisure physical activity Global Physical Activity Questionnaire Total minutes of moderate activity and double-weighted minutes of vigorous activity were summed. (Ref: [yes]) no: does not meet WHO physical activity recommendations
Body mass index	Adjustment	Continuous	The BMI is calculated using the following formula: BMI = weight (kg)/(height (m)2)	Weight (kg) Height (m)
*Work conditions*				
Presenteeism	Outcome	Categorical	Employees attend work despite physical or psychological health problems, which may impair their performance and productivity	The six-item version of the SPS (SPS-6) was used, with total scores ranging from 6 to 30. A higher score reflects greater presenteeism-related impairment.
Work sector	Prognostic factor	Categorical	Sector of activity of the participant’s company. The sector was not self-reported: it was determined based on the company name embedded in the anonymised participant ID, which includes the company identifier and a random numerical component.	No direct question. Work sector was inferred from the company associated with the participant ID. (Ref: [education]) manufacturing health care
Socio-professional category	Prognostic factor	Categorical	The socioprofessional category (CSP-2020) from INSEE (The National Institute of Statistics and Economic Studies) was used to classify workers into categories: [3]: managers and higher-level intellectual professions [4]: intermediate occupations [5]: Administrative and service workers [6]: manual workers	What is your job within your company? Socio-professional categories were defined as follows: (Ref: [white-collar]) [white-collar]: cat [3] and cat [4] from PCS-2020 classification by INSEE [blue-collar]: cat [5] and cat [6] from PCS-2020 classification by INSEE
Sedentary behaviour	Prognostic factor	Categorical	Time spent sitting or lying with low energy expenditure, while awake, in the context of occupational, educational, home and community settings, and transportation. Minutes per day	“The following question refers to the time you spend sitting or lying down at work, at home, while commuting, visiting friends, and includes time spent [sitting at a desk, travelling by car, bus, or train, reading, playing cards, or watching television], but it does not include time spent sleeping. (minutes)” Global Physical Activity Questionnaire (GPAQ) Sedentary behaviour categories were defined as follows: (Ref: [moderate]) low: ≤ 180 moderate: 180–479 high ≥ 480.
Employment status	Prognostic factor	Categorical	Employment status describing whether the participant works full-time or part-time within their company.	Do you work full-time or part-time? (Ref: [part-time])
Extended workday (10 h or more)	Prognostic factor	Categorical	Indicates whether the participant reports working days lasting 10 h or more.	Do you sometimes work shifts lasting up to 10 h per day? (Ref: [no])
Psychological demand	Prognostic factor	Categorical	Intensity and pace of workload, including time pressures, mental requirements, and task complexity. High psychological demand reflects situations in which workers are expected to sustain continuous attention, make rapid decisions, or perform under significant pressure	Job Content Questionnaire (JCQ) (Ref: [low]) high demand: > 22.5.
Decision latitude	Prognostic factor	Categorical	Degree of autonomy and skill’s discretion a worker has in their job. It combines two components: decision authority (the ability to make decisions about one’s work) and skill discretion (the opportunity to use and develop skills)	Job Content Questionnaire (JCQ) (Ref: [high]) low latitude: < 70
Social support	Prognostic factor	Categorical	Perceived level of emotional and practical assistance provided by supervisors and coworkers.	Job Content Questionnaire (JCQ) (Ref: [high]) low support: < 23,5
Job strain	Prognostic factor	Categorical	Combination of high psychological demand and low decision latitude.	Job strain categories were defined as follows: (Ref: [3]) [1]: Active (high psychological demand and high decision latitude) [2]: High strain (high psychological demand and low decision latitude) [3]: Low strain (low psychological demand and high decision latitude) [4]: Passive (low psychological demand and low decision latitude)
Iso-strain	Prognostic factor	Categorical	Simultaneous presence of job strain (high demands and low control) and low social support at work.	Iso-strain categories were defined as follows: (Ref: [3]) [1]: Iso-strain (job strain and low social support) [2] : Iso-work (no job strain and low social support) [3]: No strain (no job strain and high social support) [4]: Strain only (job strain and high social support)
*Pathology*				
Anxiety	Prognostic factor	Categorical	State of heightened psychological arousal, characterised by feelings of tension, worry, and nervousness.	Hospital Anxiety and Depression scale (HADS) Anxiety symptoms were considered present when the score on the anxiety subscale (HADS-A) was equal to or greater than 8.
Depression	Prognostic factor	Categorical	Persistent emotional state marked by low mood, loss of interest or pleasure in daily activities, and reduced motivation.	Hospital Anxiety and Depression scale (HADS) Depression symptoms were considered present when the score on the depression subscale (HADS-D) was equal to or greater than 8.
Sleep disturbances	Prognostic factor	Continuous	Disruptions in the quantity or quality of sleep that negatively affect overall sleep satisfaction	Pittsburgh Sleep Quality Index (PSQI) Score: 0–21
Number of painful sites	Prognostic factor	Continuous	Number of painful sites in the past 12 months	Section 1 Nordic questionnaire (NMQ)

### Outcome measures

All the study variables are listed in Table 1.

Presenteeism at the follow-up was assessed using the Stanford Presenteeism Scale-6 (SPS-6). We used the original instrument paper for construct definition and scoring [13] and a French translation with psychometric assessment (Lauzier, 2019), which provides item wording and equivalence procedures and compares scoring structures across French versions. Scores range from 6 to 30. For the main analysis, we created a binary outcome using cutoffs at the upper quartile of our sample (≥ 16) as a pragmatic threshold, which is consistent with prior practice in which distribution-based splits are used when validated clinical cutoffs are unavailable [13–15]. For all analyses, only presenteeism measured at the 12-month follow-up (SPS-6 score) was used as the primary outcome. Baseline SPS-6 values were not included in the analyses and were solely reported to describe the sample distribution.

### Adjustments variables

The baseline individual characteristics variables included age, gender, BMI (kg/m² of self-reported weight and height), and physical activity using the GPAQ with categorisation aligned with the WHO 2020 guidelines [16] and supported by a French GPAQ validation [17]. Physical activity compliance was defined as meeting WHO recommendations for leisure physical activity: at least 150 min of moderate-intensity activity per week spread over at least 5 days, or 75 min of vigorous-intensity activity per week spread over at least 3 days. The total minutes of moderate activity and double-weighted minutes of vigorous activity were summed to determine compliance (yes/no).

### Candidate prognostic factors

#### Work conditions

The work sector variable was derived from participants’ anonymised company identifiers, which included the company name. Each company was then classified into its corresponding activity sector, including manufacturing, education and health care.

The socioprofessional category (CSP-2020) from INSEE (The National Institute of Statistics and Economic Studies) was used to classify workers into 2 categories, white-collar workers, including managers and higher-level intellectual professions and intermediate occupations and blue-collar workers, including administrative and service employees and manual workers [18].

Employment status (full-time vs. part-time) was obtained from the question: ‘Do you work full-time or part-time?’ and extended workday were derived from the question: ‘Do you sometimes work up to 10 hours per day?’

Sedentary behaviour was assessed using the GPAQ and supported by a French GPAQ validation [17]. 

Work-related psychosocial factors were assessed using the French version of the JCQ [19, 20], which captures psychological demand (high versus ref: low), decision latitude (low versus ref: high), social support (low versus ref: high) and derived constructs. The job strain categories were defined as follows: (1) active (high psychological demand and high decision latitude), (2) high strain (high psychological demand and low decision latitude), (3) low strain (low psychological demand and high decision latitude), and (4) passive (low psychological demand and low decision latitude). The iso-strain categories combined job strain with social support levels: (1) iso-strain (job strain with low social support), (2) iso-work (no job strain with low social support), (3) no strain (no job strain with high social support), and (4) strain only (job strain with high social support).

#### Pathology

Anxiety and depression levels were assessed with the Hospital Anxiety and Depression Scale, using the validated French employee version and binary ≥ 8 clinical thresholds corresponding to having suspicion of symptoms (low, moderate or severe) [21, 22] and sleep disorders using the French version of the Pittsburgh Sleep Quality Index [23, 24].

The number of painful sites (MSDs) were assessed using the French version of the Nordic Musculoskeletal Questionnaire [21, 25] at the one-year follow-up.

### Statistical analysis

Descriptive analyses were reported using means, standard deviations and range for continuous variables and frequencies (%) for categorical data. Variables were tested for normality using the Shapiro-Wilk test in addition to visual inspection. To assess potential attrition bias, baseline characteristics were compared between participants who completed the follow-up and those lost to follow-up. Complete case analysis was used after confirming that there were no systematic differences. For continuous variables, Student’s T-Tests or Wilcoxon-Mann-Whitney tests were used according to the data distribution. For categorical variables, the chi-square test was applied. Univariate and adjusted logistic regression analyses for age, gender, and meeting WHO recommendations (leisure PA dichotomised as “meeting” vs. “not meeting” WHO activity guidelines) were used to identify candidate prognostic factors associated with high presenteeism at a follow-up. Univariate analyses were performed for all potential adjustment variables (BMI and WHO recommendations), and those with a p-value < 0.25 were considered eligible for inclusion in the adjusted analyses. Age and gender were retained irrespective of statistical significance, as these demographic characteristics are consistently used as core control covariates in empirical presenteeism research, summarised in recent systematic reviews and meta-analyses [3, 4]. To explore potential nonlinear associations between continuous candidate predictors and the outcome, we used restricted cubic splines (RCSs) in univariate and adjusted logistic regression analyses, following the framework described by Harrell (2015). Each continuous variable was tested using both a linear analysis and RCS analysis with 3, 4, and 5 knots, positioned at the recommended quantiles. The spline analysis with the lowest Akaike information criterion (AIC) was considered optimal. Additionally, likelihood ratio (LR) tests were used to formally assess whether the spline analysis provided a significantly better fit than the linear analysis. All spline curves (log-odds plots with 95% confidence intervals) were also created. Given the exploratory nature of the study, both linear and spline-based analyses are reported [26] and no formal sample size calculation was performed. Multicollinearity was assessed in the adjusted analyses using variance inflation factors (VIFs), with VIF values > 5 considered indicative of potentially problematic collinearity between adjustment variables, and > 10 severe multicollinearity, in line with established recommendations from regression diagnostics literature [27, 28]. Analyses were performed using Stata 17.0 and R version 4.4.3 (2025-02-28 ucrt) and RStudio Desktop version ‘2025.5.0.496’ with the tidyverse (v2.0.0), rms (v8.0.0), haven (v2.5.5), and ggplot2 (v3.5.2) packages.

## Results

**Fig. 1:**
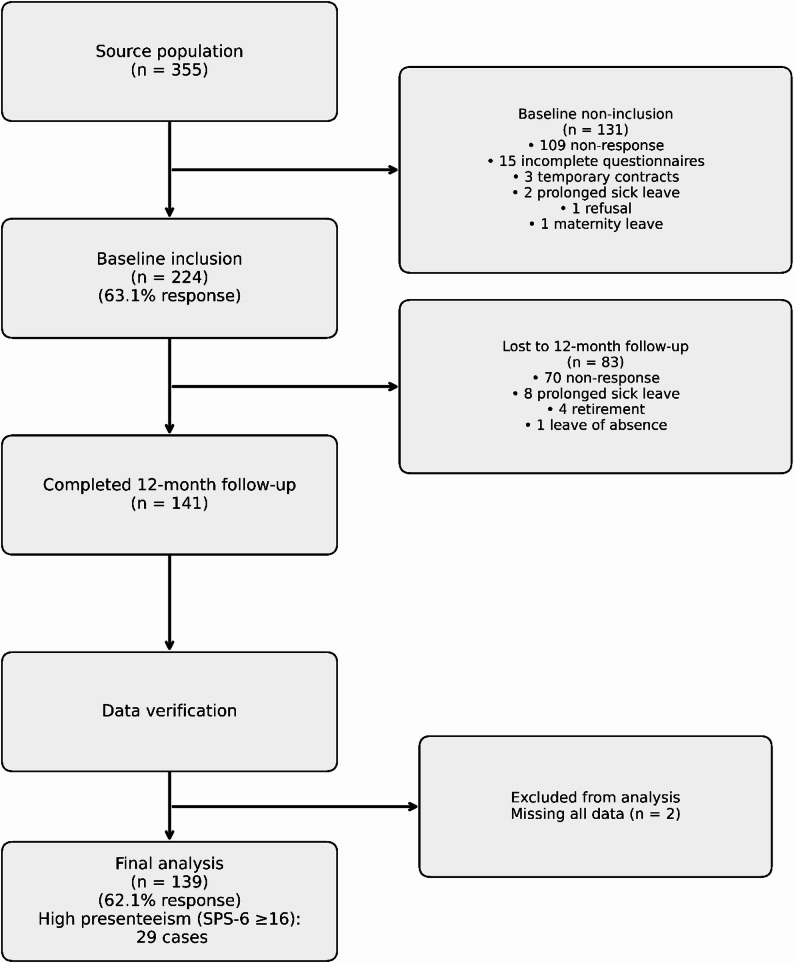
Flow diagram

Of the 355 employees invited, 224 (63.1%) completed the baseline survey. Of these 224 baseline participants, 85 (38.0%) were lost to follow-up, yielding 139 (62.1%) completers for analysis (Fig. 1).

### Baseline characteristics

At the baseline, most of the participants were women (60.7%) and the mean age was 42.9 years (± 10.8), ranging from 21 to 70 years. Participants’ mean weight at baseline was 71.3 kg (± 14.2), and the mean height was 1.69 m (± 0.09). The average BMI was 24.8 (± 4.1), and most participants were categorised as normal weight (53.1%), followed by overweight (32.1%), obese (10.7%), and underweight (4.0%). Fifty-four participants (24.1%) met the World Health Organization (WHO) recommendations for leisure-time physical activity.

Regarding work conditions, a majority of the sample comprised blue-collar occupations (54.9%), whereas 45.1% were classified as white-collar. Regarding sedentary behaviour, 32.1% of the participants reported low daily sedentary time (0–180 min/day), 36.7% reported moderate sedentary time (reference group), and 31.2% reported high sedentary time (> 479 min/day). Psychological demand averaged 22.5/36 (± 4.9), with 54.0% experiencing high psychological demand. Decision latitude averaged 70.1/96 (± 12.4), with 55.4% classified as high latitude and social support averaged 23.5/32 (± 4.0), distributed almost equally between the low (47.3%) and high (52.7%) support groups. The job strain categories (psychological demand combined with decision latitude) included active (29.5%), passive (20.1%), low strain (25.9%), and high strain (24.6%), whereas the iso-strain (job strain combined with social support) conditions revealed that most participants reported no strain (68.3%), whereas 17.0% experienced iso-strain.

For pathology, approximately 44.2% of participants (*n* = 99) presented possible anxiety symptoms and 18,7% clinical case of anxiety for the threshold at 8 and 11 from HADS, respectively. For depression, the prevalence was estimated at 17.7% and 6.7% for threshold at 8 and 11, respectively. Prevalence estimates for musculoskeletal disorders in our study population are detailed in Supplementary Table ST1.

No significant differences were observed between participants who completed the follow-up and those lost to follow-up (Table 2).

**Table 2:** Descriptive baseline data

Variable name	Missing	Total	Analysed	Lost to Follow-Up	Difference*
	*N* (%)	*N* = 224	*N* = 139	*n* = 85	*p*
*Individual’s factors*					
Age, mean ± SD [range], years	0 (0)	42.91 ± 10.79 [21–70]	42.02 ± 9.92 [21–60]	44.36 ± 12.00 [21–70]	0.14
Gender, n (%) (ref: women)	0 (0)				0.31
men		88 (39.3)	51 (36.7)	37 (43.5)	
Leisure Physical activity, n (%) (ref: yes)	0 (0)				0.87
no		170 (75.9)	106 (76.3)	64 (75.3)	
BMI, mean ± SD [range], kg/m^2^	0 (0)	24.75 ± 4.13 [16.16–40.63]	24.72 ± 4.35 [16.16–40.63]	24.81 ± 3.78 [17.30–34.99]	0.45
*Work conditions*					
Presenteeism, *n* (%) (ref: low)	0 (0)				0.65
high (> 16)		62 (27.7)	37 (26.6)	25 (29.4)	
Work sector *n* (%) (ref: education)	0 (0)				0.24
manufacturing		100 (44.6)	67 (48.2)	33 (38.8)	
health care		73 (32.6)	45 (32.4)	28 (32.9)	
Socio-professional category, *n* (%) (ref: white-collar)	0 (0)				0.06
blue-collar		123 (54.9)	83 (59.7)	40 (47.1)	
Extended workday (≥ 10 h) *n* (%) (ref: no)	0 (0)				0.27
yes		82 (36.6)	47 (33.8)	35 (41.2)	
Employment status *n* (%) (ref: part-time)	0 (0)				0.1
full-time		174 (77.7)	113 (81.3)	61 (71.8)	
Sedentary behaviour, min/day, *n* (%) (ref: moderate)	3 (1.34)				0.52
low (0–180)		71 (32.1)	40 (28.8)	31 (37.8)	
high (> 479)		69 (31.2)	47 (33.8)	22 (26.8)	
Psychological demand, *n* (%) (ref: low)	2 (0.89)				0.42
high		121 (54.0)	78 (56.1)	43 (50.6)	
Decision latitude, *n* (%) (ref: high)	2 (0.89)				0.77
low		100 (44.6)	61 (43.9)	39 (45.9)	
Social support, *n* (%) (ref: high)	2 (0.89)				0.06
low		106 (47.3)	59 (42.5)	47 (55.3)	
Job strain, *n* (%) (ref: low strain)	2 (0.89)				0.81
active		66 (29.5)	44 (31.7)	22 (25.9)	
high strain		55 (24.6)	34 (24.5)	21 (24.7)	
passive		45 (20.1)	27 (19.4)	18 (21.2)	
Iso-strain, *n* (%) (ref: no strain)	2 (0.89)				0.59
iso-strain		38 (17.0)	22 (15.8)	16 (18.8)	
iso-work		16 (7.1)	8 (5.8)	8 (9.4)	
strain only		17 (7.6)	12 (8.6)	5 (5.9)	
*Pathology*					
Anxiety, *n* (%) (ref: no)	4 (1.79)				0.12
yes		99 (44.2)	57 (41.0)	42 (51.9)	
Depression, *n* (%) (ref: no)	4 (1.79)				0.18
yes		39 (17.7)	21 (15.1)	18 (22.2)	
Sleep disturbances, mean ± SD [range]	11 (4.91)	6.57 ± 3.72 [0–18]	6.24 ± 3.56 [0–18]	7.14 ± 3.95 [0–17]	0.07
Number of painful sites (MSD), mean ± SD [range]	0 (0)	1.79 ± 1.67 [0–8]	1.89 ± 1.61 [0–6]	1.61 ± 1.77 [0–8]	0.09

^a^: WHO leisure physical activity recommendation | ^b^: sedentary at the workplace and at home

*: differences between lost to follow-up and follow up

### Outcome characteristics at follow-up

At 12-month follow-up, the mean presenteeism score was 12.3/30 (± 5.6) among the participants included in the study. Twenty-nine participants (20.9%) presented high presenteeism (SPS score > 16). Table 3 presents the distribution of individual, occupational, and clinical characteristics according to presenteeism status at 12-month follow-up and reports the statistical comparisons between participants with low presenteeism (SPS ≤ 16) and high presenteeism (SPS > 16).

**Table 3:** Baseline characteristics according to presenteeism status at 12-month follow-up

	Presenteeism		
	Low at FU	High at FU	*p* value
Individual's factors			
Age (years), median [IQR**]	42 [34–50]	42 [36–53]	0.546*
Gender (ref: women), *n* (%)			
men	44 (41.1)	6 (20.7)	**0.043**
Leisure Physical activity, *n* (%)			
no	78 (72.9)	27 (93.1)	**0.021**
BMI (kg/m^2^)(ref: ≤ 25), *n* (%)			
> 25	42 (39.3)	12 (41.4)	0.836
Work conditions			
Work sector (ref: Education), *n* (%)			**0.002**
manufacturing	56 (52.3)	11 (37.9)	
health care	28 (26.2)	17 (58.6)	
Socio-professional category (ref: white-collar), *n* (%)			
blue-collar	59 (55.1)	24 (82.8)	**0.007**
Employment status (ref: part-time), *n* (%)			
full-time	88 (82.2)	25 (86.2)	0.613
Extended workday (>10 h) (ref: no), *n* (%)			
yes	43 (40.2)	3 (10.3)	**0.003**
Sedentary behaviour (min/day) (ref: moderate [180 - 479], *n* (%)			**0.008**
low (0–180)	24 (22.4)	15 (51.7)	
high (>479)	39 (36.5)	7 (24.1)	
Psychological demand (ref: low), *n* (%)			
high	61 (57.0)	15 (51.7)	0.611
Decision latitude (ref: high), *n* (%)			
low	38 (35.5)	22 (75.9)	**0.000**
Social support (ref: high), *n* (%)			
low	41 (38.3)	16 (55.2)	0.103
Job strain (ref: low strain), *n* (%)			**0.001**
active	39 (36.5)	4 (13.8)	
high strain	22 (20.6)	11 (37.9)	
passive	16 (15.0)	11 (37.9)	
Iso-strain (ref: no strain), *n* (%)			0.199
iso-strain	13 (12.2)	8 (27.6)	
iso-work	7 (6.5)	1 (3.4)	
strain only	9 (8.4)	3 (10.3)	
Pathology			
Anxiety (ref: no), *n* (%)			
yes	41 (38.3)	15 (51.7)	0.193
Depression (ref: no), *n* (%)			
yes	12 (11.2)	9 (31.0)	**0.009**
Sleep disturbances, median [IQR**]	5 [3–9]	7 [5–9]	0.023*
Number of painful sites (MSD)at 12-month FU, median [IQR**]	**2 [1–3]**	**4 [2–4]**	0.016*

Bold: *p* value < 0.05

^a^ WHO leisure physical activity recommendation

^b^ sedentary at workplace and at home

* Mann–Whitney tests used for continuous variables with Shapiro–Wilk *p* < 0.05

** *IQR* interquartile range

### Associations between candidate prognostic factors and presenteeism

Table 4 lists the univariate and adjusted logistic regression results used to assess the linear and nonlinear associations between candidate prognostic factors and presenteeism. All variance inflation factors were below 5 (median [min - max] VIF = 1.13 [1.03–2.20]), indicating minimal multicollinearity among adjustment variables.

#### Work conditions

Among the candidates prognostic factors, work sector (health care vs. reference (education): OR, [CI 95%] = 13.96, [1.73–112.99.73.99]), socio-professional categories (blue-collar vs. reference (white-collar): OR, [CI 95%] = 3.91, [1.39–11.01]), extended workday (10 h or more) (no vs. reference (yes): OR, [CI 95%] = 0.17, [0.05–0.60]), and sedentary behaviours (low vs. reference (medium): OR, [CI 95%] = 3.93, [1.41–10.96]) were associated with high levels of presenteeism.

Decision latitude (low vs. reference (high), OR, [CI 95%] = 5.71, [2.33–15.57]), job strain (high strain vs. reference (low strain), OR, [CI 95%] = 5.00, [1.37–24.10], (passive vs. reference, OR, [CI 95%] = 6.88, [1.84–33.80])) and iso-strain (iso-strain vs. reference (no strain), OR, [CI 95%] = 5.00, [1.37–24.10]) were associated with high levels of presenteeism.

After adjustment for age, gender, and meeting the WHO recommendation for leisure PA, all associations remained statistically significant except for socio-professional categories (maintained where adjusted only for age and gender) and iso-strain.

#### Pathology

Depression (yes vs. reference (no), OR, [CI 95%] = 3.56, [1.30–9.60]), sleep disturbances (OR, [CI 95%] = 1.14, [1.01–1.27]) and the number of painful sites at FU (OR, [CI 95%] = 1.34, [1.05–1.70]) were associated with high levels of presenteeism.

After adjustment for age, gender, and meeting the WHO recommendation for leisure PA, all associations remained statistically significant except for sleep disturbances.

Sleep disturbances (spline: OR, [CI 95%] = 4.28, [2.42–7.56], spline’: OR, [CI 95%] = 5.39, [2.82–10.31]) and the number of painful sites at FU (spline: OR, [CI 95%] = 0.37, [0.15–0.87], spline’: OR, [CI 95%] = 1.02, [0.55–1.91], spline’’: OR, [CI 95%] = 2.16, [1.10–4.23]) showed a significant nonlinear association with high levels of presenteeism.

**Table 4:** Associations between presenteeism and each candidate prognostic factor in crude and adjusted linear and nonlinear logistic regression

	Crude		Adjusted*		Adjusted**	
	OR (CI 95%)	*p*	OR (CI 95%)	*p*	OR (CI 95%)	*p*
Work conditions						
Work sector (ref: Education)						
manufacturing	4.52 (0.55–37.04)	0.16	4.27 (0.50–36.16)	0.18	3.52 (0.41–30.22)	0.25
health care	**13.96 (1.73–112.99)**	**0.01**	**11.49 (1.37–96.33)**	**0.02**	**10.27 (1.21–87.48)**	**0.03**
Socio-professional category (ref: white-collar)						
blue-collar	**3.91 (1.39–11.01)**	**0.01**	**3.16 (1.01–9.85)**	**0.05**	2.88 (0.91–9.09)	0.07
Employment status (ref: part-time)						
full-time	1.35 (0.42–4.33)	0.61	1.26 (0.37–4.32)	0.71	1.08 (0.31–3.76)	0.90
Extended workday (10 h or more) (ref: no)						
yes	**0.17 (0.05–0.60)**	**0.01**	**0.18 (0.05–0.64)**	**0.01**	**0.19 (0.05–0.68)**	**0.01**
Sedentary behaviour (min/day) (ref: moderate [180–479]						
low (0–180)	**3.93 (1.41–10.96)**	**0.01**	**4.29 (1.50–12.27)**	**0.01**	**4.1 (1.42–11.87)**	**0.01**
high (> 479)	1.13 (0.36–3.50)	0.84	1.32 (0.41–4.27)	0.64	1.41 (0.43–4.57)	0.57
Psychological demand (ref: low)						
high	0.81 (0.36–1.84)	0.61	0.83 (0.35–1.95)	0.67	0.81 (0.34–1.92)	0.61
Decision latitude (ref: high)						
low	**5.71 (2.23–14.58)**	**0.00**	**5.63 (2.13–14.91)**	**0.01**	**5.52 (2.08–14.67)**	**0.01**
Social support (ref: high)						
low	1.98 (0.87–4.54)	0.11	1.99 (0.84–4.76)	0.12	2.12 (0.88–5.14)	0.12
Job strain (ref: low strain)						
active	1.03 (0.21–4.93)	0.98	1.23 (0.25–6.18)	0.80	1.21 (0.24–6.16)	0.81
high strain	**5 (1.25–20.08)**	**0.02**	**5.37 (1.24–23.32)**	0.03	**5.05 (1.16–21.90)**	**0.03**
passive	**6.88 (1.67–28.26)**	**0.01**	**7.58 (1.75–32.72)**	0.01	**7.76 (1.77–34.05)**	**0.01**
Iso-strain (ref: no strain)						
iso-strain	**2.82 (1.01–7.87)**	**0.05**	2.73 (0.92–8.08)	0.07	2.70 (0.90–8.12)	0.08
iso-work	0.66 (0.08–5.68)	0.70	0.51 (0.06–4.64)	0.55	0.59 (0.06–5.33)	0.64
strain only	1.53 (0.37–6.25)	0.55	1.30 (0.30–5.64)	0.73	1.22 (0.28–5.27)	0.80
*Pathology*						
Anxiety (ref: no)						
yes	1.72 (0.76–3.94)	0.20	1.43 (0.61–3.38)	0.41	1.54 (0.65–3.67)	0.33
Depression (ref: no)						
yes	**3.56 (1.30–9.60)**	**0.01**	**3.75 (1.33–10.61)**	**0.01**	**3.37 (1.18–9.67)**	**0.02**
Sleep disturbances	**1.14 (1.01–1.27)**	**0.03**	1.11 (0.98–1.26)	0.10	1.1 (0.97–1.25)	0.12
Sleep disturbances, 3 knots (ref < 2)		**0.05**				0.14
spline (knot = 6)	**4.28 (2.42–7.56)**		3.56 (1.63–7.77)	0.08	3.61 (0.70–18.64.70.64)	
spline’ (knot = 11)	**5.39 (2.82–10.31)**		4.25 (1.91–9.46)		3.99 (0.71–22.34)	
Number of painful sites (MSD) at 12-month FU	**1.34 (1.05–1.70)**	**0.02**	**1.33 (1.04–1.71)**	**0.02**	**1.32 (1.03–1.70)**	**0.03**
Number of painful sites (MSD) at 12-month FU 4 knots, (ref: <2)		**0.02**		**0.02**		**0.04**
spline (knot = 2)	0.37 (0.15–0.87)		0.33 (0.11–0.98)		0.34 (0.11–1.03)	
spline’ (knot = 3)	1.02 (0.55–1.91)		1.02 (0.45–2.30)		1.06 (0.47–2.41)	
spline’’ (knot = 5)	**2.16 (1.10–4.23)**		2.04 (0.90–4.63)		2.02 (0.89–4.61)	

Statistical test: logistic regression analysis and spline analysis for nonlinear model

Bold: *p* < 0.05

*CI95%* 95% confidence interval

^a^: sedentary at the workplace and at home

*: adjusted by age, gender

** : adjusted by age, gender and WHO leisure time recommendation

## Discussion

Presenteeism is a complex phenomenon at the intersection of individual vulnerability, work organisation, and broader socioeconomic pressures [1]. The strongest associations identified in this study concerned related to work factors. Low decision latitude, passive and high-strain job profiles, sedentary behaviour, extended workday, and work sector were related to higher presenteeism scores. These findings agree with recent reviews highlighting the central role of organisational stressors and hierarchical pressure in shaping presenteeism prognosis [3, 5]. Individual health indicators, multisite pain and sleep disturbances, also contributed significantly, although their effects were generally smaller than those of psychosocial exposures, consistent with prior work suggesting that behavioural and clinical factors tend to exert more modest prognostic influences relative to work-related stressors. Existing results show that pain, disability and sleep problems are associated with greater productivity losses (including components of presenteeism and absenteeism) and delayed return to full functioning [29]. Nevertheless, the literature is not fully consistent: differences in how presenteeism is conceptualised (attendance while ill vs. productivity loss) and, which instruments are used (e.g., SPS-6 vs. the World Health Organization Health and Work Performance Questionnaire or Work Limitations Questionnaire) as well as the predominance of cross-sectional over longitudinal designs, likely explain the variability in effect sizes [1, 30].

Methodological heterogeneity may partly account for the inconsistencies observed in presenteeism research. Statistical adjustment strategies vary widely across studies: while some analyses adjust only for basic demographic factors, others incorporate comprehensive sets of occupational, health, and behavioural covariates [31, 32]. Such variation can substantially influence effect size estimates and likely contributes to the inconsistent findings reported even when examining similar exposures [33]. These methodological challenges underscore the value of adopting established prognostic frameworks such as PROGRESS-2, which emphasises clarity in exposure definitions, outcome standardisation, and transparency in analytic strategies in early exploratory stages [11, 34, 35].

Furthermore, the complexity of presenteeism likely reflects residual confounding and effect modification. Associations between individual prognostic factors and presenteeism may be moderated by organisational context, cultural factors, or individual vulnerability profiles. However, these dimensions are often inadequately captured in current research designs [36, 37]. Presenteeism appears to reflect a co-occurrence of job and health features rather than isolated exposures [38]. This pattern is visible in employees facing combined low control, high demand, and poor support, who demonstrate the highest levels of presenteeism [39]. This clustering is particularly pronounced in blue-collar contexts, where low sedentary time may indicate high occupational physical load, which is consistent with the opposite pattern of occupational physical activity, resulting in sustained work-related exertion that may compromise recovery capacity [40, 41]. Musculoskeletal disorders illustrate this aggregation pattern, while isolated pain may have modest effects, multisite pain has substantially stronger associations with presenteeism, highlighting the cumulative impact of physical strain. The protective association observed for extended workday may reflect a subgroup of workers with greater decision latitude. Longer workdays in such contexts, often seen among professional, managerial or self-directed roles, may not signify increased strain, but rather flexible self-paced organisation, consistent with evidence that job control can buffer the effects of high demands.

Sleep disturbances may amplify these pathways. Although the nonlinear effects observed in crude analyses did not remain significant after adjustment, sleep disturbances remain a plausible contributor that may exacerbate the effects of both multisite pain and psychosocial stressors. Both poor sleep quality and pain independently predict reduced workplace productivity [42–44], whereas depression and anxiety further worsen these effects [45]. Convergent evidence suggests that occupational profiles characterised by high physical demands, low decision latitude, limited social support, chronic pain, and sleep disturbances represent high-risk configurations requiring targeted, multicomponent interventions rather than single factor approaches [38, 46].

### Strengths and limitations

This study is aligned with the first stage of the PROGRESS framework for prognosis research, which emphasises the exploratory identification of candidate prognostic factors through observational designs before any attempt at causal inference or validated multivariable model implementation [11, 34, 35]. The longitudinal exploratory design, with prospective follow-up and the simultaneous assessment of a broad range of variables using standardised, validated instruments, constitutes a methodological strength and enables a temporal perspective on associations.

Several limitations inherent to exploratory prognostic studies apply. The small sample size and few events limit power, widen confidence intervals, and preclude precise risk estimates. As outlined in the PROGRESS framework [11], such exploratory studies are primarily hypothesis-generating: the present findings cannot establish causality or distinguish between true prognostic and potential moderating effects. Several potential prognostic indicators identified in this analysis, such as individual factors (gender, BMI, and the WHO leisure physical activity recommendation), work conditions (socioprofessional category, sedentary behaviour, decision latitude, job strain and iso-strain), and clinical symptoms (depression, sleep disturbances, number of painful sites at the 12-month follow-up), should therefore be regarded as candidate factors requiring replication in larger, independent cohorts [11, 26, 34]. Some attrition may introduce selection bias, although baseline comparisons provide some reassurance. Missing data, which are common in longitudinal work, were mitigated by standardised follow-up but still add uncertainty [47]. The reliance on self-reported measures for key exposures and outcomes, while practical, opens the possibility of measurement and reporting biases. Furthermore, given the restricted sample size and limited the number of events, adjusting for all potential confounders in multivariable analyses was not feasible. As a result, residual confounding cannot be excluded, which limits the robustness of the conclusions. Nonlinear associations were explored via flexible regression analyses, but the small number of events likely constrained the ability to detect or confirm such patterns robustly [26]. Although MSDs were included among the candidates prognostic factors, the study was not restricted to workers with MSDs. The very high one-year prevalence of MSDs observed in this sample (92.9%) may reflect a participation bias, as symptomatic workers may have been more motivated to participate in a health-related survey [48, 49]. This could lead to an over-representation of symptomatic individuals and may limit the generalisability of the findings to the broader working population. Furthermore, given the small number of high-presenteeism cases, the statistical power was limited, and the possibility of analysis overfitting cannot be excluded [50, 51]. These findings should therefore be interpreted as hypothesis-generating, in line with the exploratory phase of the PROGRESS framework [11, 34, 35, 52].

### Implications for workplace interventions

Although the exploratory nature of this study precludes causal inference or direct recommendations, the patterns observed here may offer preliminary insights for workplace intervention. Hypothesis-generating interpretations suggest that improving psychosocial working conditions, such as increasing decision latitude, reducing job strain, and strengthening supervisor or peer support, could potentially mitigate pathways associated with higher presenteeism. On the individual side, strategies aimed at early detection and support for depression, sleep disturbances, or multisite musculoskeletal pain could complement organisational approaches. These considerations remain tentative and should not be viewed as evidence-based recommendations, but they may inform the development of multidisciplinary interventions to be tested in future confirmatory prognostic studies.

### Future research

Future studies should be prospectively registered, with sufficient sample sizes and prespecified protocols, to confirm the reproducibility and clinical utility of the candidate prognostic factors identified in this study [11, 34]. These studies should also integrate comprehensive assessments of both occupational exposure and individual vulnerability factors. The possible nonlinear associations between multisite pain, and presenteeism highlight the value of flexible statistical modelling, particularly with respect to symptom burden and work organisation [26]. Future research should specifically address potential nonlinear relationships between continuous variables and presenteeism outcomes. According to recommendations from Harrell (2015), flexible modelling strategies such as restricted cubic splines or fractional polynomial regression may offer deeper insights into complex risk patterns. Identifying thresholds or critical zones within continuous prognostic factors, such as BMI or pain intensity, could refine prognostic stratification and improve targeted intervention strategies. Given the limitations of the present study, further research should focus on external validation of these candidates’ prognostic factors in diverse occupational settings and consider the inclusion of objective measures for exposures and outcomes wherever possible.

Beyond these clinical and behavioural factors, future prognostic studies should broaden the set of organisational determinants assessed. The analyses by Böckerman & Laukkanen suggest the importance of factors such as replaceability, contractual constraints, and working-time mismatch, which were not fully explored here due to the study’s limited duration and exploratory scope [6]. Additional health conditions also warrant examination to capture the full range of potential prognostic factors.

Finally, the integration of objective measures such as activity trackers (pedometers) for physical activity, registry-based data for work absence, and clinical assessments for pain and depression should be encouraged to complement self-reported outcomes and enhance the robustness of future prognostic research.

## Conclusion

This exploratory prospective study identified several candidate prognostic factors potentially associated with high presenteeism in the working population. Given the limited sample size and the hypothesis-generating nature of these analyses, these findings should primarily inform future research.

## Supplementary Information

Supplementary Material 1.

Supplementary Material 2.

Supplementary Material 3.

Supplementary Material 4.

## Data Availability

The datasets generated and analysed during the current study are not publicly available due to French data protection regulations (CNIL) and ethical committee restrictions but are available from the corresponding author upon reasonable request and with appropriate data sharing agreements.

## Abbreviations

AIC: Akaike information criterion
ANSES: Agence nationale de sécurité sanitaire de l’alimentation, de l’environnement et du travail
BL: Baseline
BMI: Body mass index
CI: Confidence interval
CPP: Comité de Protection des Personnes
CSP: Socioprofessional category
FU: Follow-up
HADS: Hospital Anxiety and Depression Scale
JCQ: Job Content Questionnaire
MSD: Musculoskeletal Disorder
OR: Odds ratio
PROGRESS: PROGnosis RESearch Strategy
SF-12: 12-item Short Form Health Survey
SPS-6: Stanford Presenteeism Scale – 6 items
STROBE: Strengthening the Reporting of Observational Studies in Epidemiology
