# Supplementary material for: Candidate prognostic factors of presenteeism among French workers: an exploratory longitudinal study
Source: BMC Public Health. 2026 Jan 7;26:470. doi: 10.1186/s12889-025-26020-w (PMC12870923; doi:10.1186/s12889-025-26020-w)
Supplement: Supplementary file 2 — Supplementary Material 2. [file 12889_2025_26020_MOESM2_ESM.pdf]

Additional File 2

Baseline Questionnaire

Candidate Risk Factors of Presenteeism Among French Workers:
An Exploratory Longitudinal Study

INFORMATION NOTICE FOR PARTICIPANTS

Study Title

Candidate Risk Factors of Presenteeism Among French Workers:
An Exploratory Longitudinal Study

Promoter Contacts

Data Protection Officer

Coordinating Investigator

Dear Madam, Dear Sir,

You are currently working for the company XXX. Your company, together with your social and economic committee, wishes to implement a study concerning your physical, psychological and social well-being.

Objective of the Study

The objective of this research project is to determine whether you experience pain and to identify factors that could be associated with it.

Study Procedure

This study will take place in 8 stages within your company during your working hours.

Stage 1:

You will receive this information notice and must complete your non-opposition form. A participant number will be sent to you in a sealed envelope. This participant number will guarantee your anonymity throughout the study. It is important that you note it down so you know it at each stage of this study.

Stage 2:

A tablet, loaned by the Institut Franco-Européen de Chiropraxie, will be made available to you to answer a first questionnaire lasting 15 to 30 minutes depending on your responses.

This time will be included in your working hours and the computer will be installed in a location guaranteeing your anonymity.

Stage 3:
An observation of your working conditions will be carried out at your workplace, to determine gestures, postures or movements related to your pain.

Stages 4-8:
A tablet or computer will be made available to you to answer a follow-up questionnaire lasting 5 minutes each year for the next 5 years.

After this eighth stage, the study will end for you.

Benefits and Risks

- **The benefit of your participation is indirect.** Indeed, understanding factors associated with musculoskeletal pain will allow your company to consider prevention solutions for these disorders at workstations and in social life in general.
- **No compensation will be given to you** for your participation in this study.
- **This study carries no risk to your health.**

Regulatory Information

- **No reflection period** is given before providing your response regarding participation in this research. You are free to refuse or stop your participation in this study at any time without incurring any liability or prejudice and without having to justify yourself. This will not change your relationship with your company, which will not be informed of your decision. In case of study interruption, information concerning you will be kept unless you object. A question about the reason for stopping or not responding to a questionnaire will be asked in the following form: ”Why do you not wish to respond to the study or continue the study? You may choose not to answer this question.”
- **As part of this research,** a computerized processing of your personal data will be implemented to be able to meet the scientific objectives of this research, for a purpose of public interest. For this purpose, data concerning you will be transmitted to the research promoter or persons acting on their behalf, in France. These persons have signed a confidentiality agreement.
- **For data processing,** this data will be identified by your participant number transmitted at the beginning of the study. This data may also, under experimental conditions ensuring their confidentiality, be transmitted to French health authorities, to other IFEC departments.

Your Rights Regarding Personal Data

In accordance with European Regulation No. 2016/679 on Data Protection, you may:

- Request access to, rectify, receive in a readable digital format or delete data concerning you, by contacting the data protection officer.

- Object to the collection and transmission of your data or limit the use of your data only to this study or other specific situations.
- In case of disagreement, lodge a complaint with the National Commission for Information Technology and Civil Liberties (CNIL), 3 Place de Fontenoy - TSA 80715 - 75334 PARIS or at https://www.cnil.fr/webform/adresser-une-plainte

Your data will be kept until the writing of the final research report. In accordance with the decree of August 11, 2008 setting the duration of conservation by the promoter and investigator of documents and data relating to biomedical research other than that concerning medicinal products for human use, they will then be archived for at least 15 years.

This study received approval from the Ethics Committee for Research of the Committee for the Protection of Persons of [location], on [date] - No. [number].

Thanking you in advance for the trust you place in us, we remain at your disposal at ... or at the email address: ... for any additional information concerning this study.

The Research Team

Well-informed consent

I, declare that I have been well informed about the study of risk factors for musculoskeletal disorders in the workplace. I agree to participate in this study under the conditions specified above.

By clicking on the participation button, you indicate that: you have read the information notice and agree to participate.
Only one response possible.

Yes, I accept to participate

No, I do not accept

GENERAL INFORMATION (Sociodemographic informations

This first part includes general questions. Responses to these questions allow the creation of statistical groups for the study.

1. Sex *

Multiple responses possible

Female

Male

Other

2. Age *

Respond with a number between 18 and 80

____

3. Height (in m) *

Respond with a number between 1.30 and 2.60

____

4. Weight (in kg) *

Respond with a number between 30 and 200

____

5. Are you right-handed or left-handed? *

One response only

Right-handed

Left-handed

Both

6. Marital status *

One response only

Single

Married

Civil partnership (PACS)

Divorced

Widowed

Other

7. Number of dependent children *

Respond with a number

____

8. What is your job within your company? *

One response only

9. How many years of professional experience do you have? *

Respond with a number

10. Your work schedule is: *

One response only

Regular (standard 8h profile)

Variable

11. Work rhythm *

One response only

2x8h (M-E)

3x8h (N-E-M)

Weekend

Day or fixed shift

Other

(M: Morning; E: Evening; N: Night)

PHYSICAL ACTIVITY (Global Physical Activity Questionnaire)

The following questions are about the time you usually spend doing different types of physical activity. Please answer each question even if you do not consider yourself to be an active person.

Think about the time you spend doing work. Think of work as the things that you have to do such as paid or unpaid work, study/training, household chores, harvesting food/crops, fishing or hunting for food, seeking employment. In answering the following questions 'vigorous-intensity activities' are activities that require hard physical effort and cause large increases in breathing or heart rate, and 'moderate-intensity activities' are activities that require moderate physical effort and cause small increases in breathing or heart rate.

12. Does your work involve vigorous-intensity activity that causes large increases in breathing or heart rate like [carrying or lifting heavy loads, digging or construction work] for at least 10 minutes continuously? *

One response only

Yes

No → *Skip to Question 16*

13. In a typical week, on how many days do you do vigorous-intensity activities as part of your work? *

Respond with a number between 0 and 7

____

14. How much time do you spend doing vigorous-intensity activities at work on a typical day? *

Hours: Minutes

_________ : _________

15. Does your work involve moderate-intensity activity that causes small increases in breathing or heart rate such as brisk walking [or carrying light loads] for at least 10 minutes continuously? *

One response only

Yes

No → *Skip to Question 19*

16. In a typical week, on how many days do you do moderate-intensity activities as part of your work? *

Respond with a number between 0 and 7

____

17. How much time do you spend doing moderate-intensity activities at work on a typical day? *

Hours: Minutes

_________ : _________

The next questions exclude the physical activities at work that you have already mentioned.

Now think about the usual way you travel to and from places. For example to work, for shopping, to market, to place of worship. [Insert other examples if needed]

18. Do you walk or use a bicycle (pedal cycle) for at least 10 minutes continuously to get to and from places? *

One response only

Yes

No → *Skip to Question 22*

19. In a typical week, on how many days do you walk or bicycle for at least 10 minutes continuously to get to and from places? *

Respond with a number between 0 and 7

____

20. How much time do you spend walking or bicycling for travel on a typical day? *

Hours: Minutes

________ : ________

The next questions exclude the work and transport activities that you have already mentioned.

Now think about sports, fitness and recreational activities (leisure).

21. Do you do any vigorous-intensity sports, fitness or recreational (leisure) activities that cause large increases in breathing or heart rate like [running or football] for at least 10 minutes continuously? *

One response only

Yes

No → *Skip to Question 25*

22. In a typical week, on how many days do you do vigorous-intensity sports, fitness or recreational (leisure) activities? *

Respond with a number between 0 and 7

____

23. How much time do you spend doing vigorous-intensity sports, fitness or recreational activities on a typical day? *

Hours: Minutes

________ : ________

24. Do you do any moderate-intensity sports, fitness or recreational (leisure) activities that cause a small increase in breathing or heart rate such as brisk walking, [cycling, swimming, volleyball] for at least 10 minutes continuously? *

One response only

Yes

No → *Skip to Question 28*

25. In a typical week, on how many days do you do moderate-intensity sports, fitness or recreational (leisure) activities? *

Respond with a number between 0 and 7

____

26. How much time do you spend doing moderate-intensity sports, fitness or recreational (leisure) activities on a typical day? *

Hours: Minutes

_________ : _________

27. The following question refers to the time you spend sitting or lying down at work, at home, while commuting, visiting friends, and includes time spent [sitting at a desk, traveling by car, bus, or train, reading, playing cards, or watching television], but does not include time spent sleeping.

How much time do you usually spend sitting or lying down on a typical day? *

Hours: Minutes

_________ : _________

PSYCHOSOCIAL WORK FACTORS (Job Content Questionnaire)

The following questions concern your work and your working conditions.

For each question, please indicate the response that best corresponds to your situation.

Strongly disagree = 1
Disagree = 2
Agree = 3
Strongly agree = 4

28. My job requires working very fast *

1 — 2 — 3 — 4

29. My job requires working very hard *

1 — 2 — 3 — 4

30. I am not asked to do an excessive amount of work *

1 — 2 — 3 — 4

31. I have enough time to get the job done *

1 — 2 — 3 — 4

32. I am free from conflicting demands that others make *

1 — 2 — 3 — 4

33. My job requires long periods of intense concentration on the task *

1 — 2 — 3 — 4

34. My tasks are often interrupted before they can be completed, requiring attention at a later time *

1 — 2 — 3 — 4

35. My job is very hectic *

1 — 2 — 3 — 4

36. Waiting on work from other people or departments often slows me down on my job *

1 — 2 — 3 — 4

37. My job allows me to make a lot of decisions on my own *

1 — 2 — 3 — 4

38. On my job, I have very little freedom to decide how I do my work *

1 — 2 — 3 — 4

39. I have a lot of say about what happens on my job *

1 — 2 — 3 — 4

40. My job requires that I learn new things *

1 — 2 — 3 — 4

41. My job involves a lot of repetitive work *

1 — 2 — 3 — 4

42. My job requires me to be creative *

1 — 2 — 3 — 4

43. My job requires a high level of skill *

1 — 2 — 3 — 4

44. I get to do a variety of different things on my job *

1 — 2 — 3 — 4

45. I have an opportunity to develop my own special abilities *

1 — 2 — 3 — 4

46. My supervisor is concerned about the welfare of those under him/her *

1 — 2 — 3 — 4

47. My supervisor pays attention to what I am saying *

1 — 2 — 3 — 4

48. My supervisor is helpful in getting the job done *

1 — 2 — 3 — 4

49. My supervisor is successful in getting people to work together *

1 — 2 — 3 — 4

50. People I work with are competent in doing their jobs *

1 — 2 — 3 — 4

51. People I work with take a personal interest in me *

1 — 2 — 3 — 4

52. People I work with are friendly *

1 — 2 — 3 — 4

53. People I work with are helpful in getting the job done *

1 — 2 — 3 — 4

MENTAL HEALTH (Hospital Anxiety and Depression Scale

We would like to know how you are feeling. Read each item below and check the box opposite the reply that comes closest to how you have been feeling in the past week.

Don't take too long over your replies, your immediate reaction to each item will probably be more accurate than a long, thought-out response.

54. I feel tense or 'wound up' *

Most of the time

A lot of the time

From time to time, occasionally

Not at all

55. I still enjoy the things I used to enjoy *

Definitely as much

Not quite so much

Only a little

Hardly at all

56. I get a sort of frightened feeling as if something awful is about to happen *

Very definitely and quite badly

Yes, but not too badly

A little, but it doesn't worry me

Not at all

57. I can laugh and see the funny side of things *

As much as I always could

Not quite so much now

Definitely not so much now

Not at all

58. Worrying thoughts go through my mind *

A great deal of the time

A lot of the time

From time to time but not too often

Only occasionally

59. I feel cheerful *

Not at all

Not often

Sometimes

Most of the time

60. I can sit at ease and feel relaxed *

Definitely

Usually

Not often

Not at all

61. I feel as if I am slowed down *

Nearly all the time

Very often

Sometimes

Not at all

62. I get a sort of frightened feeling like 'butterflies' in the stomach *

Not at all

Occasionally

Quite often

Very often

63. I have lost interest in my appearance *

Definitely

I don't take so much care as I should

I may not take quite as much care

I take just as much care as ever

64. I feel restless as if I have to be on the move *

Very much indeed

Quite a lot

Not very much

Not at all

65. I look forward with enjoyment to things *

As much as ever I did

Rather less than I used to

Definitely less than I used to

Hardly at all

66. I get sudden feelings of panic *

Very often indeed

Quite often

Not very often

Not at all

67. I can enjoy a good book or radio or TV program *

Often

Sometimes

Not often

Very seldom

SLEEP QUALITY (Pittsburgh Sleep Quality Index)

The following questions relate to your usual sleep habits during the past month only. Your answers should indicate the most accurate reply for the majority of days and nights in the past month.

68. During the past month, when have you usually gone to bed? *

Usual bed time

____

69. During the past month, how long (in minutes) has it taken you to fall asleep each night? *

Number of minutes

____

70. During the past month, when have you usually gotten up in the morning? *

Usual getting up time

____

71. During the past month, how many hours of actual sleep do you get at night? (This may be different than the number of hours you spend in bed) *

Hours of sleep per night

____

For each of the remaining questions, check the one best response. Please answer all questions.

During the past month, how often have you had trouble sleeping because you...

72. Cannot get to sleep within 30 minutes *

Not during the past month

Less than once a week

Once or twice a week

Three or more times a week

73. Wake up in the middle of the night or early morning *

Not during the past month

Less than once a week

Once or twice a week

Three or more times a week

74. Have to get up to use the bathroom *

Not during the past month

Less than once a week

Once or twice a week

Three or more times a week

75. Cannot breathe comfortably *

Not during the past month

Less than once a week

Once or twice a week

Three or more times a week

76. Cough or snore loudly *

Not during the past month

Less than once a week

Once or twice a week

Three or more times a week

77. Feel too cold *

Not during the past month

Less than once a week

Once or twice a week

Three or more times a week

78. Feel too hot *

Not during the past month

Less than once a week

Once or twice a week

Three or more times a week

79. Have bad dreams *

Not during the past month

Less than once a week

Once or twice a week

Three or more times a week

80. Have pain *

Not during the past month

Less than once a week

Once or twice a week

Three or more times a week

81. Other reason(s), please describe: *

____

How often during the past month have you had trouble sleeping because of this? *

Not during the past month

Less than once a week

Once or twice a week

Three or more times a week

82. During the past month, how would you rate your sleep quality overall? *

Very good

Fairly good

Fairly bad

Very bad

83. During the past month, how often have you taken medicine (prescribed or ”over the counter”) to help you sleep? *

Not during the past month

Less than once a week

Once or twice a week

Three or more times a week

84. During the past month, how often have you had trouble staying awake while driving, eating meals, or engaging in social activity? *

Not during the past month

Less than once a week

Once or twice a week

Three or more times a week

85. During the past month, how much of a problem has it been for you to keep up enthusiasm to get things done? *

No problem at all

Only a very slight problem

Somewhat of a problem

A very big problem

86. Do you have a bed partner or roommate? *

No bed partner or roommate

Partner/roommate in other room

Partner in same room, but not same bed

Partner in same bed

If you have a roommate or bed partner, ask him/her how often in the past month you have had...

87. Loud snoring *

Not during the past month

Less than once a week

Once or twice a week

Three or more times a week

88. Long pauses between breaths while asleep *

Not during the past month

Less than once a week

Once or twice a week

Three or more times a week

89. Legs twitching or jerking while you sleep *

Not during the past month

Less than once a week

Once or twice a week

Three or more times a week

90. Episodes of disorientation or confusion during sleep *

Not during the past month

Less than once a week

Once or twice a week

Three or more times a week

91. Other restlessness while you sleep; please describe: *

____

Not during the past month

Less than once a week

Once or twice a week

Three or more times a week

MUSCULOSKELETAL DISORDERS (Nordic Musculoskeletal Questionnaire)

Body diagram showing different regions:

- Neck
- Shoulders
- Elbows
- Wrists/Hands
- Upper back (thoracic)
- Lower back
- Hips/Thighs
- Knees
- Ankles/Feet

92. Have you ever had problems (aches, pain, discomfort, numbness) in any body region since the beginning of your professional activity? If yes, in which region? *

This question expects a single response; it will be repeated if you have or have had another painful region

Neck

Shoulders

Elbows

Wrists/Hands

Upper back

Lower back

Hips/Thighs

Knees

Ankles/Feet

I have not had other pain

NECK PAIN SECTION

93. Have you ever had problems (aches, pain, discomfort, numbness) in the neck since the beginning of your professional activity? *

Yes

No

94. During your life: Have you ever been injured in the neck during an accident? *

Yes

No

95. During your life: Have you ever had to change jobs or tasks because of neck problems? *

Yes

No

96. During the past 12 months: Have you had any problems with your neck? *

Yes

No

97. During the past 12 months: Have you been prevented from doing your normal activities (at work or at home) because of neck problems? *

Yes

No

98. During the past 12 months: What is the total duration during which your neck problems prevented you from carrying out your usual activities (at work or at home)? *

0 days

1 to 7 days

8 to 30 days

More than 30 days

99. During the past 12 months: Have you consulted a doctor, physiotherapist, chiropractor, or any other professional for your neck problems? *

Yes

No

100. During the past 7 days: Have you had any neck problems at any time? *

Yes

No

[The questionnaire continues with identical question patterns for each body region: Shoulders, Elbows, Wrists/Hands, Upper back, Lower back, Hips/Thighs, Knees, and Ankles/Feet]

PRESENTEEISM (Stanford Presenteeism Scale-6

We would like you to describe below your professional experiences during the past month. These experiences can be affected by many environmental and personal factors and can change from time to time. For each of the following statements, please circle one of the following responses to show your agreement or disagreement with this statement.

Don't take too long over your replies, your immediate reaction to each item will probably be more accurate than a long, thought-out response.

1 if you strongly disagree with the statement
2 if you disagree with the statement
3 if you are not certain about your agreement with the statement
4 if you somewhat agree with the statement
5 if you strongly agree with the statement

101. Despite any health problems, I was able to complete the most complex tasks that my work involves. *

1 — 2 — 3 — 4 — 5

102. Despite any health problems, I managed to concentrate on achieving my goals. *

1 — 2 — 3 — 4 — 5

103. Despite any health problems, I felt that I had enough energy to complete all my work. *

1 — 2 — 3 — 4 — 5

104. Despite any health problems, I did not experience more difficulty than usual managing work-related stress. *

1 — 2 — 3 — 4 — 5

105. Despite any health problems, I experienced as much pleasure in carrying out my work. *

1 — 2 — 3 — 4 — 5

106. Despite any health problems, I felt fully capable of accomplishing all my work. *

1 — 2 — 3 — 4 — 5

FREE RESPONSE QUESTIONS

107. Suggestions for improvement to the questionnaire *

108. Do you have any suggestions for improvements to bring to your company to improve your living conditions at work? *

109. Free expression area *

We thank you for your participation in this survey.
