# Supplementary material for: Candidate prognostic factors of presenteeism among French workers: an exploratory longitudinal study
Source: BMC Public Health. 2026 Jan 7;26:470. doi: 10.1186/s12889-025-26020-w (PMC12870923; doi:10.1186/s12889-025-26020-w)
Supplement: Supplementary file 3 — Supplementary Material 3. [file 12889_2025_26020_MOESM3_ESM.pdf]

Additional File 3

12-Month Follow-up Questionnaire

Candidate Risk Factors of Presenteeism Among French Workers:
An Exploratory Longitudinal Study

PARTICIPATION CONSENT

I agree to participate in the first annual follow-up of this 5-year study within my company by checking the following box:

Yes, I agree to participate

GENERAL INFORMATION

1. Sex

Female

Male

Other

2. Height (in meters)

____

3. Weight (in kg)

____

4. Marital Status

Married

Civil partnership

Divorced

Single

Widowed

5. Your work schedule is:

Regular

Variable

(regular: standard 8h profile)

6. What is your job within your company?

One response only ______________________________________

7. Work rhythm

2x8h (M-E)

3x8h (N-E-M)

Weekend

Day or fixed shift

(M: Morning; E: Evening; N: Night; Weekend: Saturday-Sunday)

MUSCULOSKELETAL DISORDERS

Body diagram showing different regions:

- Neck
- Shoulders
- Elbows
- Wrists/Hands
- Upper back (thoracic)
- Lower back
- Hips/Thighs
- Knees
- Ankles/Feet

Have you ever had problems (aches, pain, discomfort, numbness) in any body region since the beginning of your professional activity? If yes, in which region? *

This question expects a single response; it will be repeated if you have or have had another painful region

Neck

Shoulders

Elbows

Wrists/Hands

Upper back

Lower back

Hips/Thighs

Knees

Ankles/Feet

I have not had other pain

NECK PAIN SECTION

Have you ever had problems (aches, pain, discomfort, numbness) in the neck since the beginning of your professional activity? *

Yes

No

During your life: Have you ever been injured in the neck during an accident? *

Yes

No

During your life: Have you ever had to change jobs or tasks because of neck problems? *

Yes

No

During the past 12 months: Have you had any problems with your neck? *

Yes

No

During the past 12 months: Have you been prevented from doing your normal activities (at work or at home) because of neck problems? *

Yes

No

During the past 12 months: What is the total duration during which your neck problems prevented you from carrying out your usual activities (at work or at home)? *

0 days

1 to 7 days

8 to 30 days

More than 30 days

During the past 12 months: Have you consulted a doctor, physiotherapist, chiropractor, or any other professional for your neck problems? *

Yes

No

During the past 7 days: Have you had any neck problems at any time? *

Yes

No

[The questionnaire continues with identical question patterns for each body region: Shoulders, Elbows, Wrists/Hands, Upper back, Lower back, Hips/Thighs, Knees, and Ankles/Feet]

PRESENTEEISM

We would like you to describe below your professional experiences during the past month. These experiences can be affected by many environmental and personal factors and can change from time to time. For each of the following statements, please circle one of the following responses to show your agreement or disagreement with this statement.

1 if you strongly disagree with the statement
2 if you disagree with the statement
3 if you are not certain about your agreement with the statement
4 if you somewhat agree with the statement
5 if you strongly agree with the statement

Despite the pain mentioned above, I was able to complete the most complex tasks that my work involves.

1 — 2 — 3 — 4 — 5

Despite the pain mentioned above, I managed to concentrate on achieving my goals.

1 — 2 — 3 — 4 — 5

Despite the pain mentioned above, I felt that I had enough energy to complete all my work.

1 — 2 — 3 — 4 — 5

Despite the pain mentioned above, I did not experience more difficulty than usual managing work-related stress.

1 — 2 — 3 — 4 — 5

Despite the pain mentioned above, I experienced as much pleasure in carrying out my work.

1 — 2 — 3 — 4 — 5

Despite the pain mentioned above, I felt fully capable of accomplishing all my work.

1 — 2 — 3 — 4 — 5
