# Supplementary material for: Candidate prognostic factors of presenteeism among French workers: an exploratory longitudinal study
Source: BMC Public Health. 2026 Jan 7;26:470. doi: 10.1186/s12889-025-26020-w (PMC12870923; doi:10.1186/s12889-025-26020-w)
Supplement: Supplementary file 4 — Supplementary Material 4. [file 12889_2025_26020_MOESM4_ESM.pdf]

Musculoskeletal disorders	Baseline (N=224)	Follow-up 1 (N=139)
	n (%) (CI95%)	n (%) (CI95%)
Sites		
Low back	91 (40,6) (34,1–47,4)	71 (51,1) (42,5–59,6)
Neck pain	80 (35,7) (29,4–42,4)	69 (49,6) (41,1–58,2)
Shoulder	59 (26,3) (20,7–32,6)	51 (36,7) (28,7–45,3)
Thoracic	53 (23,7) (18,3–29,8)	42 (30,2) (22,7–38,6)
Wrist/Hand	37 (16,5) (11,9–22,0)	35 (25,2) (18,2–33,2)
Knee	20 (8,9) (5,5–13,5)	24 (17,3) (11,4–24,6)
Ankle/Foot	17 (7,6) (4,5–11,9)	18 (12,9) (7,9–19,7)
Hip	20 (8,9) (5,5–13,5)	17 (12,2) (7,3–18,9)
Elbow	23 (10,3) (6,6–15,0)	15 (10,8) (6,2–17,2)
No pain	59 (26,3) (20,7–32,6)	16 (7,1) (4,1–11,3)
At least 1 pain	165 (73,7) (67,4–79,3)	208 (92,9) (88,7–95,9)
Multisite 1*	157 (70,1) (63,6–76,0)	79 (56,8) (48,2–65,2)
Multisite 2*	50 (22,3) (17,0–28,3)	44 (31,7) (24,0–40,1)
Multisite 3*	17 (7,6) (4,5–11,9)	16 (11,5) (6,7–18,0)

Multisite 1 : 0-2 painful sites at FU1 | Multisite 2 : 3-4 painful sites at FU1

Multisite 3 : 5 or more painful sites at FU1

*Categories depending on spline analysis

CI95% : 95% Confidence Interval
